# Proteomic analysis of S-nitrosylation induced by 1-methyl-4-phenylpyridinium (MPP^+^)

**DOI:** 10.1186/1477-5956-10-74

**Published:** 2012-12-29

**Authors:** Akira T Komatsubara, Tomoya Asano, Hiroki Tsumoto, Kazuharu Shimizu, Takumi Nishiuchi, Masanori Yoshizumi, Kentaro Ozawa

**Affiliations:** 1 Department of Pharmacology, Nara Medical University School of Medicine, 840 Shijo-cho, Kashihara, Nar, 634-8521, Japan; 2 Department of Genomic Drug Discovery Science, Kyoto University Graduate School of Pharmaceutical Sciences, Sakyo-ku, Kyoto, Japan; 3 Division of Functional Genomics, Advanced Science Research Center, Kanazawa University, Kanazawa, Japan; 4 World-Leading Drug Discovery Research Center, Kyoto University, Sakyo-ku, Kyoto, Japan; 5 Department of Nanobio Drug Discovery, Kyoto University Graduate School of Pharmaceutical Sciences, Sakyo-ku, Kyoto, 606-8501, Japan

## Abstract

**Background:**

Nitric oxide (NO) mediates its function through the direct modification of various cellular targets. S-nitrosylation is a post-translational modification of cysteine residues by NO that regulates protein function. Recently, an imbalance of S-nitrosylation has also been linked to neurodegeneration through the impairment of pro-survival proteins by S-nitrosylation.

**Results:**

In the present study, we used two-dimensional gel electrophoresis in conjunction with the modified biotin switch assay for protein S-nitrosothiols using resin-assisted capture (SNO-RAC) to identify proteins that are S-nitrosylated more intensively in neuroblastoma cells treated with a mitochondrial complex I inhibitor, 1-methyl-4-phenylpyridinium (MPP^+^). We identified 14 proteins for which S-nitrosylation was upregulated and seven proteins for which it was downregulated in MPP^+^-treated neuroblastoma cells. Immunoblot analysis following SNO-RAC confirmed a large increase in the S-nitrosylation of esterase D (ESD), serine-threonine kinase receptor-associated protein (STRAP) and T-complex protein 1 subunit γ (TCP-1 γ) in MPP^+^-treated neuroblastoma cells, whereas S-nitrosylation of thioredoxin domain-containing protein 5 precursor (ERp46) was decreased.

**Conclusions:**

These results suggest that S-nitrosylation resulting from mitochondrial dysfunction can compromise neuronal survival through altering multiple signal transduction pathways and might be a potential therapeutic target for neurodegenerative diseases.

## Background

Neurodegenerative diseases, including Parkinson’s, Alzheimer’s and Huntington’s disease, occur as a result of the progressive loss of the structure or function of neurons, including the death of neurons. The exact mechanism of neuronal cell death in neurodegeneration is not fully understood, but it is usually accompanied by protein aggregation [1,2] and increased indices of oxidative stress [3].

S-nitrosylation is the post-translational modification (PTM) of the thiol group of a cysteine residue by nitric oxide (NO). Recently, this process has been receiving much attention as a mechanism by which NO ubiquitously influences cellular signal transduction, including neurodegeneration [4]. Recent studies have suggested that nitrosative stress due to the generation of excessive NO can mediate excitotoxicity in part by triggering protein misfolding and aggregation via the S-nitrosylation of protein disulfide isomerase (PDI) or the E3 ubiquitin ligase parkin [5]. S-nitrosylation of PDI inhibits the activity of this enzyme, which leads to the accumulation of misfolded proteins, activation of the unfolded protein response, and neuronal cell death triggered by protein misfolding and aggregation [6]. Parkin, mutation of which causes a form of autosomal recessive juvenile Parkinson disease, was also reported to be S-nitrosylated and S-nitrosylation inhibits the E3 ubiquitin ligase activity and protective function of this protein [7].

S-nitrosylation is also involved in oxidative stress produced by mitochondria, which can subsequently induce neurodegeneration [3]. One of the major sources of oxidative stress in cells is the process of oxidative phosphorylation in the mitochondria, and NO can mediate excitotoxicity in part by triggering mitochondrial fragmentation via S-nitrosylation, in the absence of genetic predisposition. S-nitrosylation of the mitochondrial fission protein dynamin-related protein 1 (Drp1) is activated by amyloid-β peptide, which is a key mediator of the pathogenesis of Alzheimer’s disease. The resulting hyperactivation of Drp1 leads to excessive mitochondrial fragmentation, the production of oxidative stress, and neuronal cell death [8].

In different neurodegenerative disorders, the abnormal programmed cell death of neurons has been suggested to be one of the major causes of the degeneration, and the S-nitrosylation of glyceraldehyde 3-phosphate dehydrogenase (GAPDH) triggers cell death in many types of cell [9]. GAPDH catalyzes the sixth step of glycolysis and thus serves to break down glucose for energy and carbon molecules. However, over the years, there have been reports that GAPDH is implicated in several non-metabolic processes, including the initiation of apoptosis. NO causes the S-nitrosylation of GAPDH, followed by translocating the complex of GAPDH and the E3 ubiquitin ligase Siah to the nucleus and subsequent cell death [9,10].

In the present study, to investigate the effects of S-nitrosylation on protein function under conditions of mitochondrial dysfunction, we analyzed the level of S-nitrosylated proteins in the neuroblastoma cell line SH-SY5Y after exposure to the mitochondrial complex I inhibitor 1-methyl-4-phenylpyridinium (MPP^+^). Using the two-dimensional electrophoresis (2DE) technique, we identified several protein spots for which the intensity was increased by exposure to MPP^+^. We then evaluated the S-nitrosylation of several proteins that were identified by immunoblotting after separation by the modified biotin switch assay for protein S-nitrosothiols using resin-assisted capture (SNO-RAC) [11].

## Results and discussion

### Purification of S-nitrosylated proteins by SNO-RAC

S-nitrosylation plays important roles in modifying the function of proteins under physiological and pathophysiological conditions [4], and accurate quantification of the extent of S-nitrosylation at a particular cysteine residue is essential to understand its influence on signal transduction. One method used for quantification is the biotin-switch technique (BST), in which S-nitrosylated cysteines are converted into more stable biotinylated forms [12]. The BST method involves three steps: 1) free cysteine thiols are blocked with S-methylmethanethiosulfonate, 2) nitrosylated cysteines are reduced by ascorbate without the concomitant reduction of disulfide bonds or other oxidative cysteine PTMs, and 3) newly-exposed cysteine thiols are alkylated classically by biotin-HPDP. However, other techniques, such as SNO-RAC, have also been employed to good effect [11].

**Figure 1:**
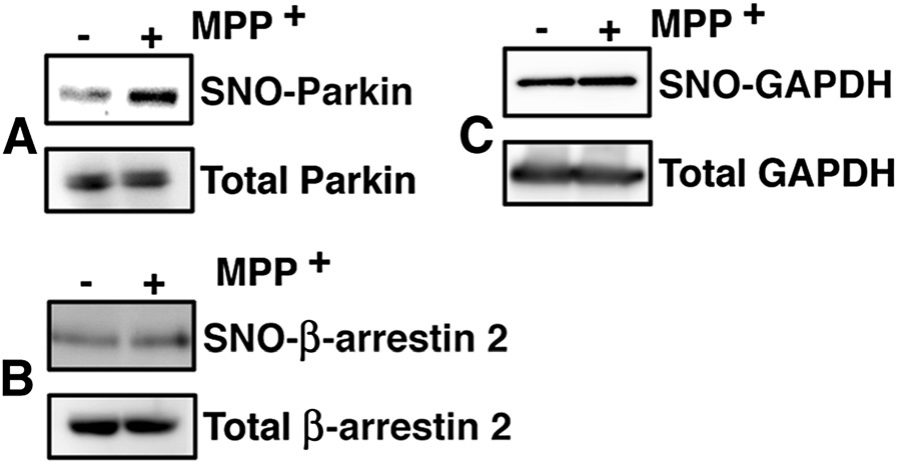
**S-nitrosylation of parkin, β-arrestin 2, and GAPDH in neuroblastoma cells treated with MPP**^**+**^**.** Parkin **(A)** or β-arrestin 2 **(B)** were overexpressed in SH-SY5Y cells. The SH-SY5Y cells were then treated with MPP^+^ (100 μM) for 3 h, and lysates prepared from the treated cells were analyzed by the SNO-RAC assay, in which ascorbate-dependent capture of proteins by resin indicates the presence of S-nitrosylated cysteine residues. Elutants were subjected to immunoblot analysis using anti-FLAG **(A, B)** or anti-GAPDH **(C)** antibody.

We tested that MPP^+^ enhances S-nitrosylation nonspecifically or not by evaluating levels of S-nitrosylated proteins which have been reported, Parkin, β-arrestin 2 and GAPDH. Parkin [7] and GAPDH [9] were reported to be S-nitrosylated more potently under MPP^+^-treatment, whereas S-nitrosylation of β-arrestin 2 is increased by the activation of GPCR and denitrosylated rapidly [13]. To detect S-nitrosylated parkin or β-arrestin 2, parkin and β-arrestin 2 were overexpressed separately in SH-SY5Y cells. After the cells had been treated with MPP^+^ for 3 h, we purified the S-nitrosylated proteins by SNO-RAC from cell extracts that had been prepared. Then we performed immunoblot analysis using anti-FLAG (to detect overexpressed parkin (Figure 1A) or β-arrestin 2 (Figure 1B) and anti-GAPDH (Figure 1C) antibodies, respectively. Consistent with previous studies, the level of S-nitrosylated parkin was increased in MPP^+^-treated cells as compared with control cells. In contrast, the levels of S-nitrosylated β-arrestin 2 and GAPDH were not altered. S-nitrosylation of GAPDH was reported to be up-regulated in brain tissues exposed to 1-methyl-4-phenyl-1,2,3,6-tetrahydropyridine (MPTP) [14], which is metabolized into MPP^+^ in brain tissue, which suggests that the overexpression of inducible NO synthase (iNOS) as a result of inflammation might contribute significantly to S-nitrosylation of GAPDH in brain tissues treated with MPTP. On the other hand, Chung et al. reported Parkin was S-nitrosylated in brain tissue of iNOS knockout mice treated with MPTP [7], suggesting nNOS contribute to S-nitrosylation of Parkin induced by MPTP. The results obtained in the present study showed that treatment with MPP^+^ does not upregulate the S-nitrosylation of all proteins.

### Proteomic analysis of S-nitrosylated proteins in SH-SY5Y cells

**Figure 2:**
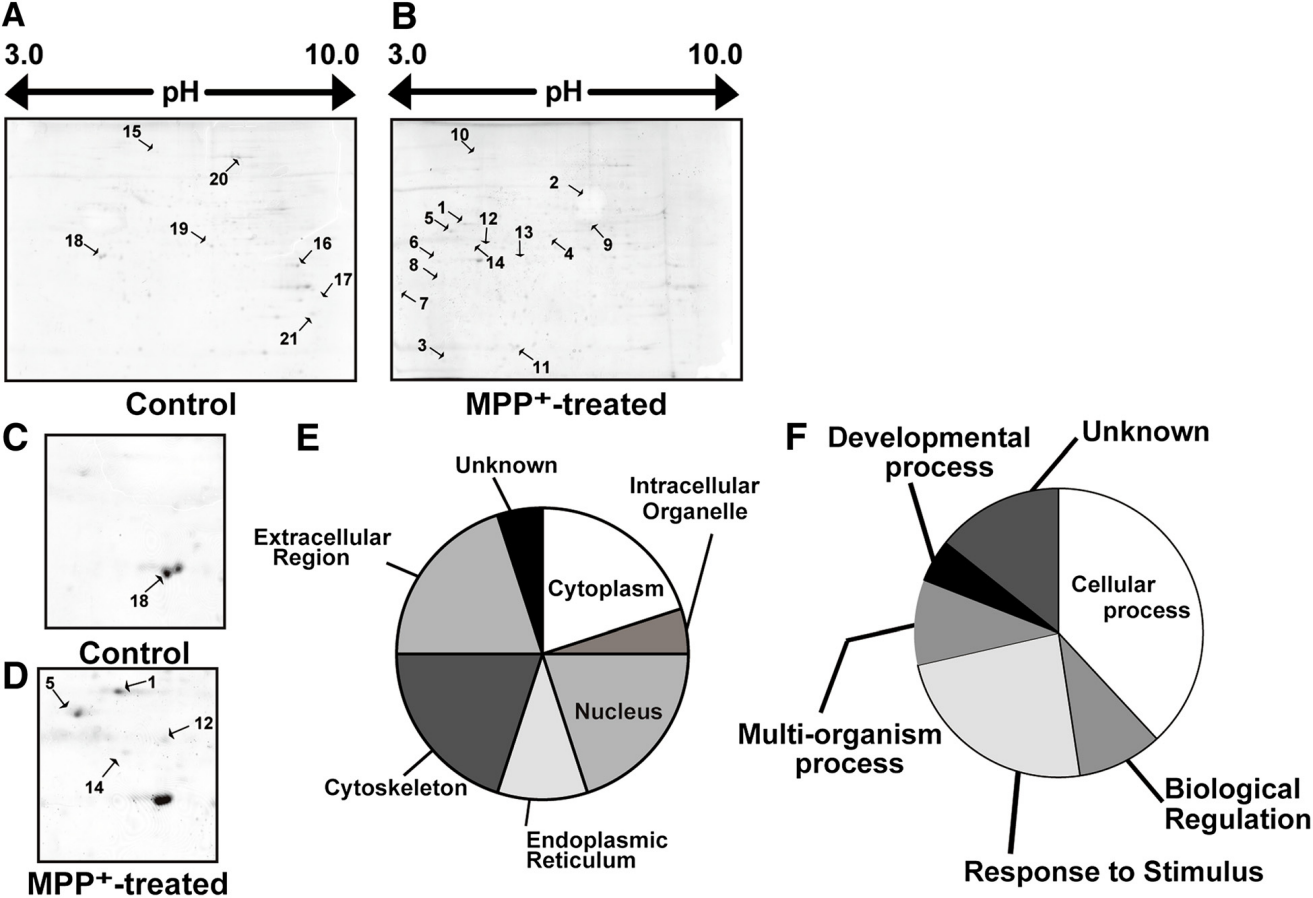
**2D-gel electrophoresis and categorization of the proteins.** Representative 2D images of S-nitrosylated proteins in the cell extracts of control **(A)** and MPP + -treated cells **(B)**. High-magnification images of S-nitrosylated proteins in the cell extracts of control **(C)** and MPP^+^-treated cells **(D)**. Number represents ID of each spot. The differentially expressed proteins can be classified into 7 biological processes **(E)**, and 6 cellular localization sites **(F).**

To investigate the roles of S-nitrosylation in MPP^+^-treated neuroblastoma cells, we performed 2DE as described in the Materials and Methods. From the 2DE, we identified several protein spots for which the levels differed between control neuroblastoma cells and cells treated with MPP^+^ (Figure 2A, B, C and D). The spots were picked, and the proteins in the picked gel plugs were digested in gel with trypsin and prepared for mass spectrometry analysis as described in the Materials and Methods.

Fourteen proteins were identified from 14 spots of which the signals increased in MPP^+^-treated cells, whereas another set of seven proteins were identified from seven spots of which the signals decreased in MPP^+^-treated cells. These upregulated and downregulated S-nitrosylated proteins are listed in Tables 1 and 2, respectively, together with their accession number, signal-ratio (MPP^+^-treated/control), name, calculated molecular weight, calculated pI, the ProtScore and sequence coverage. We also analyzed the results of mass spectrometry using Scaffold version 3.0 (Proteome Software, Portland, OR) and found that S-nitrosylation of proteins belonging to a wide range of functional categories was altered in samples from MPP^+^-treated cells (Figure 2E and F).

**Table 1:** Identified proteins uniquely increased in MPP + -treated cells compared with control

**SPOT #**	**Accession**	**Ratio (MPP+/Control)**	**NAME**	**Mass (kDa)**	**pI**	**ProtScore (a)**	**% Cov (b)**
**1**	**TM11E_HUMAN**	**1.78**	**Transmembrane protease, serine 11E precursor/DESC1**	**47.7**	**8.85**	**1.4**	**5.2**
	**TBA6_HUMAN**		**Tubulin alpha-6 chain**	**49.9**	**4.96**	**12**	**16.7**
**2**	**TM11E_HUMAN**	**2.15**	**Transmembrane protease, serine 11E precursor/DESC1**	**47.7**	**8.85**	**1.7**	**4**
**3**	**TM11E_HUMAN**	**1.85**	**Transmembrane protease, serine 11E precursor/DESC1**	**47.7**	**8.85**	**0.7**	**3.3**
**4**	**TM11E_HUMAN**	**1.82**	**Transmembrane protease, serine 11E precursor/DESC1**	**47.7**	**8.85**	**0.64**	**3.3**
	**TNF10_HUMAN**		**Tumor necrosis factor ligand superfamily member 10**	**32.5**	**7.01**	**1.05**	**5.3**
**5**	**TBB2C_HUMAN**	**1.92**	**Tubulin beta-2C chain**	**49.8**	**4.79**	**15.74**	**23.4**
	**TBB5_HUMAN**		**Tubulin beta chain**	**49.7**	**4.78**	**15.74**	**23.4**
**6**	**RSSA_HUMAN**	**3.45**	**40S ribosomal protein SA/Laminin receptor**	**32.9**	**4.79**	**6**	**18**
**7**	**PCNA_HUMAN**	**1.51**	**Proliferating cell nuclear antigen (PCNA)**	**28.8**	**4.57**	**10**	**32.6**
**8**	**STRAP_HUMAN**	**2.14**	**Serine-threonine kinase receptor-associated protein**	**38.4**	**4.98**	**2**	**2.9**
**9**	**TCPG_HUMAN**	**2.45**	**T-complex protein 1 subunit gamma**	**60.5**	**6.1**	**14.22**	**22**
**10**	**GBLP_HUMAN**	**1.69**	**Guanine nucleotide-binding protein subunit beta 2-like 1**	**35.1**	**7.6**	**2**	**1.2**
**11**	**ESTD_HUMAN**	**2.15**	**S-formylglutathione hydrolase/Esterase D**	**31.5**	**6.54**	**9.7**	**25.5**
**12**	**KNG1_HUMAN**	**3.87**	**kininogen-1 precursor**	**72**	**6.34**	**2**	**1.2**
**13**	**TXND5_HUMAN**	**1.52**	**Thioredoxin domain-containing protein 5 precursor/ERp46**	**47.6**	**5.63**	**3.55**	**9.5**
**14**	**FIBB_HUMAN**	**2.45**	**Fibrinogen beta chain precursor**	**55.9**	**8.54**	**2**	**2.9**

a. The Total ProtScore is a measure of the total amount of evidence for a detected protein, which was calculated using all of the peptides detected for the protein.

b. % Sequence coverage.

**Table 2:** Identified proteins uniquely decreased in MPP + -treated cells compared with control

**SPOT #**	**Accession**	**Ratio (MPP+/Control)**	**NAME**	**Mass (kDa)**	**pI**	**ProtScore (a)**	**% Cov (b)**
**15**	**GANAB_HUMAN**	**0.57**	**Neutral alpha-glucosidase AB precursor**	**106.9**	**5.74**	**4.25**	**6.1**
**16**	**PRD13_HUMAN**	**0.65**	**PR domain zinc finger protein 13**	**74**	**9**	**2.01**	**4.2**
**17**	**ROA2_HUMAN**	**0.58**	**Heterogeneous nuclear ribonucleoproteins A2/B1**	**36**	**8.67**	**3.71**	**10.8**
**18**	**ACTG_HUMAN**	**0.66**	**Actin, cytoplasmic 2**	**41.8**	**5.31**	**24**	**38.7**
**19**	**EF1G_HUMAN**	**0.46**	**Elongation factor 1-gamma**	**50.1**	**6.25**	**16.74**	**23.6**
**20**	**EF2_HUMAN**	**0.59**	**Elongation factor 2**	**95.3**	**6.41**	**18.45**	**25.8**
**21**	**FHL1_HUMAN**	**0.64**	**Four and a half LIM domain protein 1**	**36.3**	**9.25**	**15.7**	**23.5**

a. The Total ProtScore is a measure of the total amount of evidence for a detected protein, which was calculated using all of the peptides detected for the protein.

b. % Sequence coverage.

Our results suggested S-nitrosylation might play an important role in DNA repair. MPP^+^-treatment enhanced S-nitrosylation of proliferating cell nuclear antigen (PCNA), which plays an important role in several DNA damage-responsive pathways as well as DNA replication [15]. In additon, PCNA was reported to be associated with Parkin [16], suggesting S-nitrosylation of PCNA might involve the regulation of DNA repair caused by mitochondrial dysfunction. Furthermore, MPP^+^-treatment enhanced S-nitrosylation of STRAP, which activates p53 by removing Mdm2, a negative regulator of p53 [17], suggesting S-nitrosylation of STRAP might regulate DNA repair through the activation of p53.

S-nitrosylation of the α- and β-subunit of tubulin, which have been reported to be S-nitrosylated previously [12], was upregulated in MPP^+^-treated neuroblastoma cells, which suggested that our experiment detected S-nitrosylated proteins. In a previous report, S-nitrosylation of the α- and β-subunit of tubulin was shown to regulate their polymerization by regulating the formation of inter- and intramolecular disulfide bonds [18].

### Detection of S-nitrosylated proteins in SH-SY5Y cells

To confirm the results of the 2DE, S-nitrosylation of esterase D (ESD), serine-threonine kinase receptor-associated protein (STRAP), T-complex protein 1 subunit γ (TCP-1 γ), and thioredoxin domain-containing protein 5 precursor (ERp46) were analyzed. ESD, which is also known as S-formylglutathione hydrolase (FGH), constitutes part of a formaldehyde detoxification pathway by catalyzing the hydrolysis of S-formylglutathione to formic acid and glutathione [19]. ESD was first purified from human tissues, in which polymorphisms in its expression were associated with several diseases, including retinoblastoma [20] and Wilson’s disease [21]. STRAP is a transforming growth factor-β (TGF-β) receptor-interacting protein that inhibits TGF-β signaling by stabilizing the association between TGF-β receptors and SMAD7 [22,23]. TCP-1 γ is a molecular chaperone that is a member of the chaperonin-containing TCP1 complex (CCT), which is also known as the TCP1 ring complex (TRiC) [24]. ERp46 has a PDI domain that exhibits a high sequence similarity to thioredoxin, which catalyzes the rate limiting reaction of disulphide bond formation, isomerization, and reduction [25].

**Figure 3:**
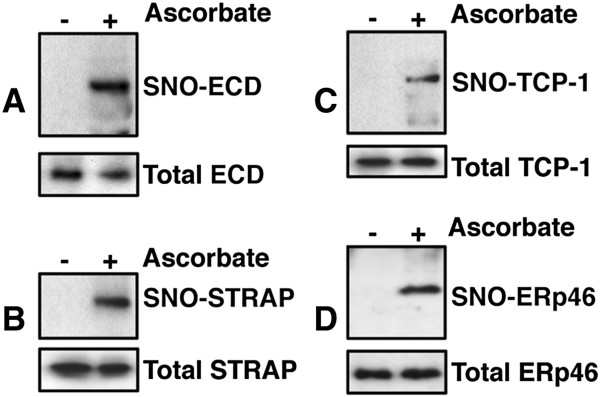
**Detection of S-nitrosylated ESD, STRAP, TCP-1 γ, and ERp46.** ESD **(A)**, STRAP **(B)**, TCP-1 γ **(C)**, and ERp46 **(D)** were overexpressed in SH-SY5Y cells, and lysates prepared from the treated cells were analyzed by the SNO-RAC assay, in which ascorbate-dependent capture of proteins by resin indicates the presence of S-nitrosylated cysteine residues. Elutants were subjected to immunoblot analysis using anti-FLAG antibody.

We amplified and subcloned cDNA for ESD, STRAP, TCP-1 γ, and ERp46 with a FLAG tag. Then we analyzed cell extracts prepared from SH-SY5Y cells overexpressing ESD, STRAP, TCP-1, and ERp46, respectively, by SNO-RAC assay, followed by immunoblot analysis using an anti-FLAG antibody (Figure 3). The samples that were prepared in the absence of ascorbate showed negligible signals, which demonstrated that each protein contained cysteine(s) that were reduced in an ascorbate-dependent manner. The results of the assay showed that ESD, STRAP, TCP-1, and ERp46 were S-nitrosylated in SH-SY5Y cells in the absence of MPP^+^ treatment.

**Figure 4:**
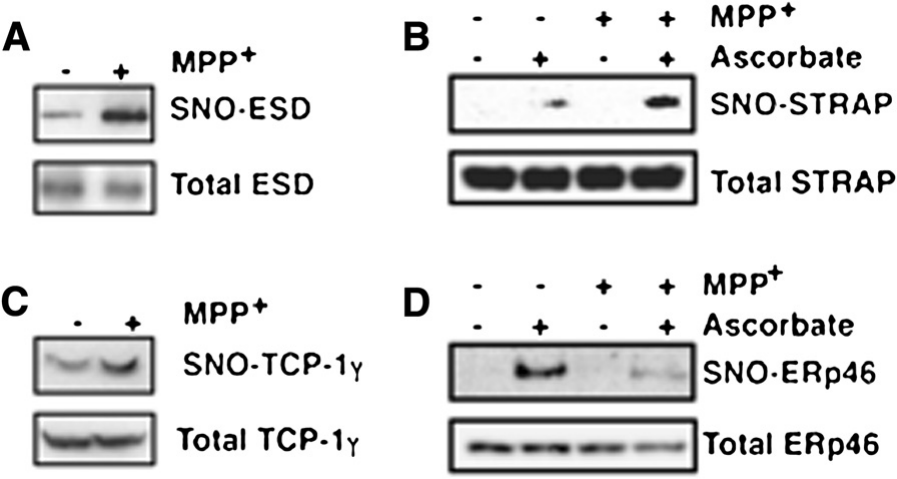
**S-nitrosylation of ESD, STRAP, TCP-1 γ, and ERp46 in neuroblastoma cells treated with MPP**^**+**^**.** ESD **(A)**, STRAP **(B)**, TCP-1 γ **(C)**, and ERp46 **(D)** were overexpressed in SH-SY5Y cells. The SH-SY5Y cells were then treated with MPP^+^ (100 μM) for 3 h, and lysates prepared from the treated cells were analyzed by the SNO-RAC assay, in which ascorbate-dependent capture of proteins by resin indicates the presence of S-nitrosylated cysteine residues. Elutants were subjected to immunoblot analysis using anti-FLAG antibody.

Furthermore, we evaluated levels of S-nitrosylated proteins in cell extracts that had been prepared from MPP^+^-treated (3 h) SH-SY5Y cells by the SNO-RAC assay. Consistent with our 2DE experiment, treatment with MPP^+^ increased the level of S-nitrosylation of ESD, STRAP, and TCP-1, which indicated that our proteomics method that combined SNO-RAC and 2DE successfully detected proteins that show enhanced S-nitrosylation upon exposure to MPP^+^ (Figure 4A, C and D). On the other hand, S-nitrosylation of ERp46 was decreased by MPP^+^-treatment (Figure 4B), possibly because of electrophoretic mobility shift by other PTM(s) in ERp46 or existence of protein(s) in the gel-plug.

### Concluding remarks

In the present study, we used a combination of 2DE and SNO-RAC to identify S-nitrosylated proteins that had been modified more intensively than others upon exposure to MPP^+^. We confirmed that ESD, STRAP and TCP-1 were S-nitrosylated markedly upon MPP^+^ treatment, whereas S-nitrosylation of ERp46 decreased. Our observations provided clear evidence that MPP^+^ treatment changed the level of S-nitrosylation on several proteins, which were involved in multiple signal transduction pathways. This suggests that the neurotoxicity of MPP^+^ is effected through multiple pathways and S-nitrosylation might play an important role in this process. It is likely that application of the experimental approach described in the present study in other cell and biological systems will provide a more comprehensive understanding of the precise role of S-nitrosylation in the stress response or the pathogenesis of diseases.

## Materials and methods

### Materials

Sequencing-grade trypsin was purchased from Promega (Madison, WI). The protease inhibitor cocktail was obtained from Roche Applied Science (Switzerland). IPG buffer was purchased from GE Healthcare, and ZipTipC18 columns from Millipore, respectively. Anti-FLAG monoclonal antibody and 1-methyl-4-phenylpyridinium was obtained from Sigma-Aldrich (St. Louis, MO).

### Transfection

Transfection of the expression constructs were performed using lipofectamine LTX (Invitrogen).

### Purification of S-nitrosylated proteins by SNO-RAC

S-nitrosylated proteins were purified by using SNO-RAC [11] with some modifications. Briefly, SNO-RAC Resins were prepared as described. Then, lysates (250 ml diluted with 750 ml HEN buffer containing 250 mM HEPES, 1 mM EDTA, 0.1 mM neocuproine [pH 8.0]) were incubated with SDS (1% final concentration) and methyl methanethiosulfonate (Sigma-Aldrich; St. Louis, MO) at 50°C for 25 min. Proteins were precipitated with acetone, washed three times, resuspended in HENS buffer (HEN containing 1% SDS; 200 ml). This material is added to 50 μl resin slurry in the presence of sodium ascorbate (Fluka, final 20 mM). Following rotation in the dark for 3 h, the resin was washed with 4 x 1 ml HENS buffer. Captured proteins are eluted with 30 ml HENS buffer containing 100 mM 2- mercaptoethanol for 20 min at RT, precipitated with acetone and resuspended in sample buffer (4 M urea, 2%(w/v) CHAPS, 10 mg/ml DTT and 1%(v/v) PharmalyteTM) .

### Separation by 2D-gel electrophoresis

2DE was carried out as reported previously [26]. In brief, for isoelectric focusing (IEF), samples were loaded onto rehydration strips that were 18 cm in length with an immobilized pH gradient from 3 to 10 and separated on a MultiPhor Unit (GE Healthcare). After the first separation, the strips were equilibrated in equilibration buffer A [50 mM Tris–HCl, pH 8.8, 6 M urea, 30% (v/v) glycerol, 2% (w/v) SDS, 1% DTT, 0.002% (w/v) bromophenol blue] and then in equilibration buffer B [50 mM Tris–HCl, pH 8.8, 6 M urea, 30% (v/v) glycerol, 2% (w/v) SDS, 2.5% iodoacetamide, 0.002% (w/v) bromophenol blue] for 15 min. Separation in the second dimension was achieved using a Hoefer SE 600 Ruby electrophoresis system (GE Healthcare) and 10% polyacrylamide gels. After electrophoresis, gels were stained with Coomassie Brilliant Blue (Figure 1). The experiments were performed twice and the similar images were obtained.

### Quantitation analysis

After staining, gels were scanned and analyzed by ImageJ (National Institutes of Health, Bethesda, MD) to detect, subtract background of, normalize, and quantify the spots in images from a single 2D-gel. Our quantitation analysis revealed the signal intensities of 14 spots were ≥1.5-fold higher (i.e., increase in protein expression) and signal intensities of 7 spots were 2/3-fold lower (i.e., decrease in protein expression) in the MPP^+^-treated sample compared to their respective spots in the wild-type sample.

### Identification of proteins

Proteins were identified as described previously [26]. MS/MS analysis was performed with a 4800 plus MALDI TOF/TOFTM Analyzer (ABsciex, California, USA), and proteins were identified by using the ParagonTM algorithm [27] of the Protein PilotTM software (ABsciex, California, USA). For each protein identification, total ProtScore were used. The total ProtScore is a measurement of all the peptide evidence for a protein and is analogous to protein scores reported by other protein identification software. Basically proteins whose ProtScores were higher than 1.0 were listed in Tables 1 and 2, but Transmembrane protease, serine 11E precursor/DESC1 with lower ProtScore was anomalistically included in the Table 1 because DESC1 was identified from several spots. Cellular localizations and biological processes of proteins identified by MIS were categorized using Scaffold version 3.0 (Proteome Software, Portland, OR).

### Western blotting

Cell lysates were prepared using a Qproteome Mammalian Protein Prep Kit (Qiagen, Germany). Samples were analyzed by western blotting. After transfer to PVDF membranes, immunoreactive bands were visualized using the enhanced chemiluminescence detection system and indicated antibodies as described previously [28].

## Abbreviations

Smad: Sma- and mad-related protein; IPG: Immobilized pH gradient; HPDP: N-(6-(Biotinamido)hexyl)-3′-(2′-pyridyldithio)-propionamide.

## Competing interests

The authors declare no conflict of interest in relation to this work.

## Authors’ contributions

TA & KO designed the study. ATK, TA & KO performed the work. TA & KO interpreted the data. KO wrote the manuscript, and TA, HT, KS, TN & MY helped to draft the manuscript. All authors read and approved the final manuscript.
